# Supplementary material for: Changes in HIV incidence during the COVID-19 pandemic (2020–22) compared with the pre-pandemic period (2015–19) in Peru: An observational study
Source: PLoS One. 2025 Jun 2;20(6):e0324784. doi: 10.1371/journal.pone.0324784 (PMC12129149; doi:10.1371/journal.pone.0324784)
Supplement: S2 Table — (DOC) [file pone.0324784.s002.doc]

**Table S2. HIV incidence by sex and age** in Peru, 2015–2019

|  | **2015** | | | | **2016** | | | | **2017** | | | | **2018** | | | | | **2019** | | |
| --- | --- | --- | --- | --- | --- | --- | --- | --- | --- | --- | --- | --- | --- | --- | --- | --- | --- | --- | --- | --- |
|  | **Population** | **Cases** | **IR** | **(95% CI)** | **Population** | **Cases** | **IR** | **(95% CI)** | **Population** | **Cases** | **IR** | **(95% CI)** | **Population** | **Cases** | **IR** | **(95% CI)** | **Population** | **Cases** | **IR** | **(95% CI)** |
| **Total** | 31,151,643 | 7781 | 24.98 | 24.43-25.54 | 31,488,628 | 7664 | 24.34 | 23.80-24.89 | 31,826,018 | 6876 | 21.60 | 21.10-22.12 | 32,162,184 | 8452 | 26.28 | 25.72-26.85 | 32,526,084 | 8141 | 25.03 | 24.48-25.58 |
| **Sex** |  |  |  |  |  |  |  |  |  |  |  |  |  |  |  |  |  |  |  |  |
| Male | 15,605,814 | 5900 | 37.81 | 36.85-38.78 | 15,772,388 | 5957 | 37.77 | 36.82-38.74 | 15,939,059 | 5281 | 33.13 | 32.24-34.04 | 16,105,008 | 6655 | 41.32 | 40.34-42.33 | 16,013,654 | 6336 | 39.57 | 38.60-40.55 |
| Female | 15,545,829 | 1878 | 12.08 | 11.54-12.64 | 15,716,240 | 1707 | 10.86 | 10.35-11.39 | 15,886,959 | 1594 | 10.03 | 9.55-10.54 | 16,057,176 | 1797 | 11.19 | 10.68-11.72 | 16,512,430 | 1805 | 10.93 | 10.43-11.45 |
| **Age group**  **(years)** |  |  |  |  |  |  |  |  |  |  |  |  |  |  |  |  |  |  |  |  |
| 0–11 | 6,952,948 | 139 | 2.00 | 1.68-2.36 | 6,921,937 | 129 | 1.86 | 1.56-2.21 | 6,888,014 | 70 | 1.02 | 0.79-1.28 | 6,852,187 | 58 | 0.85 | 0.64-1.09 | 6,817,540 | 94 | 1.38 | 1.11-1.69 |
| 12–17 | 3,482,425 | 157 | 4.51 | 3.83-5.27 | 3,482,327 | 143 | 4.11 | 3.46-4.84 | 3,484,064 | 156 | 4.48 | 3.80-5.24 | 3,486,220 | 161 | 4.62 | 3.93-5.39 | 3,489,507 | 162 | 4.65 | 3.96-5.41 |
| 18–29 | 6,640,669 | 3296 | 49.63 | 47.65-51.36 | 6,676,681 | 3317 | 49.68 | 48.00-51.40 | 6,704,876 | 3065 | 45.71 | 44.11-47.36 | 6,728,020 | 3927 | 58.37 | 56.56-60.22 | 6,757,135 | 3659 | 54.15 | 52.41-55.93 |
| 30–59 | 11,064,551 | 3913 | 35.37 | 34.27-36.49 | 11,289,378 | 3818 | 33.82 | 32.76-34.91 | 11,519,188 | 3369 | 29.25 | 28.27-30.25 | 11,750,205 | 4041 | 34.39 | 33.34-35.47 | 11,993,916 | 3960 | 33.01 | 31.99-34.05 |
| ≥60 | 3,011,050 | 273 | 9.07 | 8.02-10.21 | 3,118,305 | 257 | 8.24 | 7.26-9.31 | 3,229,876 | 213 | 6.59 | 5.74-7.54 | 3,345,552 | 265 | 7.92 | 7.00-8.93 | 3,467,986 | 265 | 7.65 | 6.75-8.62 |

Abbreviations: IR, incidence rate; CI, confidence interval.
