# Supplementary material for: Changes in HIV incidence during the COVID-19 pandemic (2020–22) compared with the pre-pandemic period (2015–19) in Peru: An observational study
Source: PLoS One. 2025 Jun 2;20(6):e0324784. doi: 10.1371/journal.pone.0324784 (PMC12129149; doi:10.1371/journal.pone.0324784)
Supplement: S3 Table — (DOC) [file pone.0324784.s003.doc]

**Table S3. HIV incidence in among the regions of Peru, 2015–2019**

|  | **2015** | | | | **2016** | | | | **2017** | | | | **2018** | | | | **2019** | | | |
| --- | --- | --- | --- | --- | --- | --- | --- | --- | --- | --- | --- | --- | --- | --- | --- | --- | --- | --- | --- | --- |
|  | **Population** | **Cases** | **IR** | **(95% CI)** | **Population** | **Cases** | **IR** | **(95% CI)** | **Population** | **Cases** | **IR** | **(95% CI)** | **Population** | **Cases** | **IR** | **(95% CI)** | **Population** | **Cases** | **IR** | **(95% CI)** |
| Amazonas | 422 629 | 218 | 51.58 | 44.96-58.90 | 423,898 | 202 | 47.65 | 41.31-54.70 | 424,952 | 259 | 60.95 | 53.75-68.84 | 425,829 | 221 | 51.90 | 45.28-59.21 | 427,041 | 221 | 51.85 | 45.15-59.04 |
| Ancash | 1,148,634 | 112 | 9.75 | 8.03-11.73 | 1,154,639 | 151 | 13.08 | 11.07-15.34 | 1,160,490 | 116 | 10.00 | 8.26-11.99 | 1,166,182 | 137 | 11.75 | 9.86-13.89 | 1,171,756 | 137 | 11.73 | 9.82-13.82 |
| Apurimac | 458 830 | 3 | 0.65 | 0.13-1.91 | 460,868 | 7 | 1.52 | 0.61-3.13 | 462,791 | 5 | 1.08 | 0.35-2.52 | 464,584 | 12 | 2.58 | 1.33-4.51 | 466,936 | 10 | 2.10 | 1.03-3.94 |
| Arequipa | 1,287,205 | 279 | 21.67 | 19.21-24.37 | 1,301,298 | 324 | 24.90 | 22.26-27.76 | 1,315,528 | 320 | 24.32 | 21.73-27.14 | 1,329,802 | 354 | 26.62 | 23.92-29.54 | 1,350,676 | 324 | 23.96 | 21.45-26.75 |
| Ayacucho | 688,657 | 27 | 3.92 | 2.58-5.70 | 696,152 | 34 | 4.88 | 3.38-6.82 | 703,629 | 51 | 7.25 | 5.40-9.53 | 711,058 | 76 | 10.69 | 8.42-13.38 | 718,634 | 51 | 7.12 | 5.28-9.33 |
| Cajamarca | 1,529,755 | 51 | 3.33 | 2.48-4.38 | 1,533,783 | 46 | 3.00 | 2.20-4.00 | 1,537,172 | 56 | 3.64 | 2.75-4.73 | 1,540,004 | 71 | 4.61 | 3.60-5.82 | 1,543,104 | 62 | 3.99 | 3.08-5.15 |
| Callao | 1,010,315 | 628 | 62.16 | 57.39-67.22 | 1,024,439 | 878 | 85.71 | 80.13-91.57 | 1,038,706 | 613 | 59.02 | 54.44-63.88 | 1,053,029 | 694 | 65.91 | 61.09-71.00 | 1,067,815 | 706 | 66.15 | 61.33-71.18 |
| Cusco | 1,316,729 | 68 | 5.16 | 4.01-6.55 | 1,324,371 | 87 | 6.57 | 5.26-8.10 | 1,331,758 | 99 | 7.43 | 60.4-9.05 | 1,338,898 | 161 | 12.02 | 10.24-14.03 | 1,346,373 | 120 | 8.90 | 7.39-10.66 |
| Huancavelica | 494,963 | 13 | 2.63 | 1.40-4.49 | 498,556 | 12 | 2.41 | 1.24-4.20 | 502,084 | 23 | 4.58 | 2.90-6.87 | 505,498 | 16 | 3.17 | 1.81-5.14 | 509,117 | 17 | 3.26 | 1.95-5.35 |
| Huanuco | 860,537 | 87 | 10.11 | 0.81-12.47 | 866,631 | 69 | 7.96 | 6.19-10.08 | 872,523 | 102 | 11.69 | 9.53-14.19 | 878,199 | 100 | 11.39 | 9.26-13.85 | 889,134 | 93 | 10.50 | 8.44-12.81 |
| Ica | 787,170 | 277 | 35.19 | 31.17-39.59 | 794,919 | 234 | 29.44 | 25.79-33.46 | 802,610 | 202 | 25.17 | 21.82-28.89 | 810,213 | 232 | 28.63 | 25.07-32.57 | 817,965 | 237 | 28.93 | 25.4-32.91 |
| Junin | 1,350,783 | 189 | 13.99 | 12.07-16.14 | 1,360,506 | 76 | 5.59 | 4.40-6.99 | 1,370,274 | 130 | 9.49 | 7.93-11.27 | 1,379,937 | 183 | 13.26 | 11.41-15.33 | 1,389,850 | 161 | 11.58 | 9.86-13.52 |
| La Libertad | 1,859,640 | 304 | 16.35 | 14.56-18.29 | 1,882,405 | 338 | 17.96 | 16.09-19.98 | 1,905,301 | 368 | 19.31 | 17.39-21.39 | 1,928,197 | 563 | 29.20 | 26.84-31.71 | 1,956,389 | 415 | 21.21 | 19.22-23.35 |
| Lambayeque | 1,260,650 | 279 | 22.13 | 19.61-24.89 | 1,270,794 | 323 | 25.42 | 22.72-28.35 | 1,280,788 | 184 | 14.37 | 12.37-16.60 | 1,290,617 | 221 | 17.12 | 14.94-19.54 | 1,300,720 | 254 | 19.54 | 17.20-22.08 |
| Lima | 9,838,251 | 3840 | 39.03 | 37.81-40.29 | 9,989,369 | 3415 | 34.19 | 33.05-35.35 | 10,143,003 | 2790 | 27.51 | 26.50-28.55 | 10,298,159 | 3536 | 34.34 | 33.21-35.49 | 10,458,367 | 3614 | 34.55 | 33.44-35.70 |
| Loreto | 1,039,372 | 486 | 46.76 | 42.69-51.11 | 1,049,364 | 498 | 47.46 | 43.38-51.81 | 1,058,946 | 530 | 50.05 | 45.88-54.50 | 1,068,132 | 560 | 52.43 | 48.18-56.96 | 1,077,831 | 569 | 52.83 | 48.54-57.31 |
| Madre de Dios | 137,316 | 59 | 42.97 | 32.71-55.42 | 140,508 | 61 | 43.41 | 33.21-55.77 | 143,687 | 78 | 54.28 | 42.91-67.75 | 146,856 | 91 | 61.97 | 49.89-76.08 | 150,181 | 78 | 51.94 | 41.05-64.82 |
| Moquegua | 180,477 | 23 | 12.74 | 8.08-19.12 | 182,333 | 42 | 23.03 | 16.60-31.14 | 184,187 | 39 | 21.17 | 15.06-28.95 | 186,036 | 45 | 24.19 | 17.64-32.37 | 187,941 | 39 | 20.96 | 14.76-28.37 |
| Pasco | 304,158 | 5 | 1.64 | 0.53-3.84 | 306,322 | 1 | 0.33 | 0.01-1.82 | 308,465 | 6 | 1.95 | 0.71-4.23 | 310,578 | 15 | 4.83 | 2.70-7.97 | 312,777 | 8 | 2.62 | 1.10-5.04 |
| Piura | 1,844,129 | 145 | 7.86 | 6.64-9.25 | 1,858,620 | 175 | 9.42 | 8.07-10.92 | 1,873,024 | 238 | 12.71 | 11.14-14.43 | 1,887,210 | 293 | 15.53 | 13.80-17.41 | 1,901,896 | 257 | 13.49 | 11.91-15.27 |
| Puno | 1,415,608 | 17 | 1.20 | 0.70-1.92 | 1,429,098 | 69 | 4.83 | 3.76-6.11 | 1,442,930 | 74 | 5.13 | 4.03-6.44 | 1,456,989 | 86 | 5.90 | 4.72-7.29 | 1,471,405 | 64 | 4.35 | 3.35-5.55 |
| San Martin | 840,790 | 210 | 24.98 | 21.71-28.59 | 851,883 | 151 | 17.73 | 15.01-20.79 | 862,822 | 159 | 18.43 | 15.67-21.53 | 873,593 | 199 | 22.78 | 19.72-26.17 | 884,795 | 194 | 21.88 | 18.95-25.24 |
| Tacna | 341,838 | 86 | 25.16 | 10.12-31.07 | 346,013 | 86 | 24.85 | 19.88-30.70 | 350,105 | 78 | 22.28 | 17.61-27.81 | 354,158 | 116 | 32.75 | 27.07-39.28 | 358,314 | 102 | 28.36 | 23.21-34.56 |
| Tumbes | 237,685 | 66 | 27.77 | 21.48-35.33 | 240,590 | 42 | 17.46 | 12.58-23.60 | 243,362 | 61 | 25.07 | 19.17-32.20 | 246,050 | 76 | 30.89 | 24.34-38.66 | 248,877 | 69 | 27.89 | 21.57-35.09 |
| Ucayali | 495,522 | 304 | 61.35 | 54.65-68.65 | 501,269 | 339 | 67.63 | 60.62-75.22 | 506,881 | 295 | 58.20 | 51.75-65.23 | 512,376 | 393 | 76.70 | 69.30-84.67 | 518,190 | 337 | 65.03 | 58.28-72.36 |

Abbreviations: IR, incidence rate; CI, confidence interval.
