# Supplementary material for: Changes in HIV incidence during the COVID-19 pandemic (2020–22) compared with the pre-pandemic period (2015–19) in Peru: An observational study
Source: PLoS One. 2025 Jun 2;20(6):e0324784. doi: 10.1371/journal.pone.0324784 (PMC12129149; doi:10.1371/journal.pone.0324784)
Supplement: Attachment — Submitted filename: PONE-D-25-07972_reviewer_ic.pdf [file pone.0324784.s004.pdf]

PLOS ONE

Changes in HIV incidence during the COVID-19 pandemic compared with pre-pandemic Peru: an observational study

--Manuscript Draft--

**Manuscript Number:**	PONE-D-25-07972
**Article Type:**	Research Article
**Full Title:**	Changes in HIV incidence during the COVID-19 pandemic compared with pre-pandemic Peru: an observational study
**Short Title:**	Changes in HIV incidence during the COVID-19 pandemic compared with pre-pandemic Peru

**Corresponding Author:**	Max Carlos Ramírez-Soto, BSc, MPH, PhD, FRSPH<br>Universidad Tecnologica del Peru<br>Lima, PERU

**Keywords:**	HIV infection; COVID-19; pandemic; epidemiology

Abstract:

Introduction

During the COVID-19 pandemic, non-pharmaceutical interventions affected the screening of sexually transmitted infections. We investigated the incidence of HIV infection during the COVID-19 pandemic compared with incidence in the pre-pandemic period.

Methods

In this observational study, we analyzed HIV surveillance data for all age-groups from 25 geographically diverse from Peru from Jan 1, 2015 to Dec 31, 2022. HIV incidence during the COVID-19 pandemic years (2020, 2021, and 2022) were compared with pre-pandemic rates (2015–19).

Results

Overall, there were 65 166 new cases of HIV infection from January 1, 2015 to December 31, 2022. HIV incidence risk ratio (IRR) was 26% lower in 2020 (IRR=0.74; 95% CI, 0.71–0.76), 5% higher in 2021 (IRR=1.05; 95% CI, 1.02–1.08) and 16% higher in 2022 (IRR=1.16; CI, 1.13-1.20), compared with the pre-pandemic period. By 2022. Furthermore, compared with the pre-pandemic period, the annual incidence of HIV among men was 29% lower in 2020 (IRR = 0.71; 95% CI, 0.68–0.73), and 4% higher in 2021 (IRR=1.04, 95% CI, 1.01-1.08) and 10% higher in 2022 (IRR=1.10; 95% CI, 1.06–1.14). In the age stratified analysis, the annual HIV incidence in 2020 was 21 and 33% lower for those aged 18-29 (IRR=0.79; 95% CI, 0.75–0.83) and 30-59 (IRR=0.67; 95% CI, 0.64–0.70), respectively, compared with the pre-pandemic period. Finally, annual HIV incidence has decreased in 11 out of 24 regions in 2020, compared with the pre-pandemic period.

Conclusions

Our study showed that during the COVID-19 pandemic in 2020, the incidence of HIV infection in the population of Peru was decreased. However, this incidence started to revert to pre-pandemic rates in 2021, which was temporally associated with the reduction or elimination of non-pharmaceutical interventions. By 2022, the incidence of HIV infection was higher to the pre-pandemic period especially in regions of the Peruvian Amazon.

**Order of Authors:**	Max Carlos Ramírez-Soto, BSc, MPH, PhD, FRSPH
	Hugo Arroyo-Hernández

**Additional Information:**	
**Question**	**Response**

Powered by Editorial Manager® and ProduXion Manager® from Aries Systems Corporation

**Financial Disclosure**<br><br>Enter a financial disclosure statement that describes the sources of funding for the work included in this submission. Review the submission guidelines for detailed requirements. View published research articles from *PLOS ONE* for specific examples.<br><br>This statement is required for submission and **will appear in the published article** if the submission is accepted. Please make sure it is accurate.<br><br>**Funded studies**<br>Enter a statement with the following details:<br>• Initials of the authors who received each award<br>• Grant numbers awarded to each author<br>• The full name of each funder<br>• URL of each funder website<br>• Did the sponsors or funders play any role in the study design, data collection and analysis, decision to publish, or preparation of the manuscript?<br><br>Did you receive funding for this work?	The author(s) received no specific funding for this work.
**Competing Interests**<br><br>Use the instructions below to enter a competing interest statement for this submission. On behalf of all authors, disclose any competing interests that could be perceived to bias this work—acknowledging all financial support and any other relevant financial or non-financial competing interests.<br><br>This statement is required for submission and **will appear in the published article** if the submission is accepted. Please make sure it is accurate and that any funding sources listed in your Funding Information later in the submission form are also declared in your Financial Disclosure statement.	The authors have declared that no competing interests exist.

View published research articles from *PLOS ONE* for specific examples.<br><br>**NO authors have competing interests**<br><br>Enter: *The authors have declared that no competing interests exist.*<br><br>**Authors with competing interests**<br><br>Enter competing interest details beginning with this statement:<br><br>*I have read the journal's policy and the authors of this manuscript have the following competing interests: [insert competing interests here]*<br><br>* typeset	
**Ethics Statement**<br><br>Enter an ethics statement for this submission. This statement is required if the study involved:<br><br>• Human participants<br>• Human specimens or tissue<br>• Vertebrate animals or cephalopods<br>• Vertebrate embryos or tissues<br>• Field research<br><br>Write "N/A" if the submission does not require an ethics statement.<br><br>General guidance is provided below. Consult the submission guidelines for detailed instructions. **Make sure that all information entered here is included in the Methods section of the manuscript.**	N/A

**Format for specific study types**<br><br>**Human Subject Research (involving human participants and/or tissue)**<br>• Give the name of the institutional review board or ethics committee that approved the study<br>• Include the approval number and/or a statement indicating approval of this research<br>• Indicate the form of consent obtained (written/oral) or the reason that consent was not obtained (e.g. the data were analyzed anonymously)<br><br>**Animal Research (involving vertebrate animals, embryos or tissues)**<br>• Provide the name of the Institutional Animal Care and Use Committee (IACUC) or other relevant ethics board that reviewed the study protocol, and indicate whether they approved this research or granted a formal waiver of ethical approval<br>• Include an approval number if one was obtained<br>• If the study involved *non-human primates*, add *additional details* about animal welfare and steps taken to ameliorate suffering<br>• If anesthesia, euthanasia, or any kind of animal sacrifice is part of the study, include briefly which substances and/or methods were applied<br><br>**Field Research**<br><br>Include the following details if this study involves the collection of plant, animal, or other materials from a natural setting:<br>• Field permit number<br>• Name of the institution or relevant body that granted permission	
**Data Availability**<br><br>Authors are required to make all data underlying the findings described fully available, without restriction, and from the time of publication. PLOS allows rare exceptions to address legal and ethical concerns. See the PLOS Data Policy and FAQ for detailed information.	Yes - all data are fully available without restriction

A Data Availability Statement describing where the data can be found is required at submission. Your answers to this question constitute the Data Availability Statement and **will be published in the article**, if accepted.<br><br>**Important:** Stating 'data available on request from the author' is not sufficient. If your data are only available upon request, select 'No' for the first question and explain your exceptional situation in the text box.<br><br>Do the authors confirm that all data underlying the findings described in their manuscript are fully available without restriction?	
**Describe where the data may be found in full sentences. If you are copying our sample text, replace any instances of XXX with the appropriate details.**<br><br>• If the data are **held or will be held in a public repository**, include URLs, accession numbers or DOIs. If this information will only be available after acceptance, indicate this by ticking the box below. For example: *All XXX files are available from the XXX database (accession number(s) XXX, XXX.*).<br>• If the data are all contained **within the manuscript and/or Supporting Information files**, enter the following: *All relevant data are within the manuscript and its Supporting Information files.*<br>• If neither of these applies but you are able to provide **details of access elsewhere**, with or without limitations, please do so. For example:<br><br>*Data cannot be shared publicly because of [XXX]. Data are available from the XXX Institutional Data Access / Ethics Committee (contact via XXX) for researchers who meet the criteria for access to confidential data.*<br><br>*The data underlying the results presented in the study are available from (include the name of the third party*	Data used as input into these analyses are publicly available at https://www.dge.gob.pe/vih/#grafico02.

and contact information or URL).<br>• This text is appropriate if the data are owned by a third party and authors do not have permission to share the data.<br><br>* typeset	
Additional data availability information:	

Manuscript

Click here to access/download;Manuscript;Manuscript HIV_Pendemic revHA.docx

Changes in HIV incidence during the COVID-19 pandemic compared with pre-pandemic Peru: an observational study

Max Carlos Ramírez-Soto [1,*] and Hugo Arroyo-Hernández [2]

Author Affiliations:

1 Facultad de Ciencias de la Salud, Universidad Tecnológica del Peru, Lima, Peru

2 Instituto Nacional de Salud, Lima, Peru

***Corresponding author:** maxcrs22@gmail.com

Funding: The authors have not declared a specific grant for this research from any funding agency in the public, commercial or not-for-profit sectors.

Declaration of competing interest: The authors declare that no competing interests exist.

Ethics approval: This study was approved by the Institutional Review Board (Ethics Research Committee) of Universidad Tecnologica del Peru (Lima, Peru).

ABSTRACT

Introduction. During the COVID-19 pandemic, non-pharmaceutical interventions affected the screening of sexually transmitted infections. We investigated the incidence of HIV infection during the COVID-19 pandemic compared with incidence in the pre-pandemic period.

Methods. In this observational study, we analyzed HIV surveillance data for all age-groups from 25 geographically diverse from Peru from Jan 1, 2015 to Dec 31, 2022. HIV incidence during the COVID-19 pandemic years (2020, 2021, and 2022) were compared with pre-pandemic rates (2015–19).

Results. Overall, there were 65 166 new cases of HIV infection from January 1, 2015 to December 31, 2022. HIV incidence risk ratio (IRR) was 26% lower in 2020 (IRR=0.74; 95% CI, 0.71–0.76), 5% higher in 2021 (IRR=1.05; 95% CI, 1.02–1.08) and 16% higher in 2022 (IRR=1.16; CI, 1.13-1.20), compared with the pre-pandemic period. By 2022. Furthermore, compared with the pre-pandemic period, the annual incidence of HIV among men was 29% lower in 2020 (IRR = 0.71; 95% CI, 0.68–0.73), and 4% higher in 2021 (IRR=1.04, 95% CI, 1.01-1.08) and 10% higher in 2022 (IRR=1.10; 95% CI, 1.06–1.14). In the age stratified analysis, the annual HIV incidence in 2020 was 21 and 33% lower for those aged 18-29 (IRR=0.79; 95% CI, 0.75–0.83) and 30-59 (IRR=0.67; 95% CI, 0.64–0.70), respectively, compared with the pre-pandemic period. Finally, annual HIV incidence has decreased in 11 out of 24 regions in 2020, compared with the pre-pandemic period.

Conclusions. Our study showed that during the COVID-19 pandemic in 2020, the incidence of HIV infection in the population of Peru was decreased. However, this incidence started to revert to pre-pandemic rates in 2021, which was temporally associated with the reduction or elimination of non-pharmaceutical interventions. By 2022, the incidence of HIV infection was higher to the pre-pandemic period especially in regions of the Peruvian Amazon.

Keywords: HIV infection; COVID-19; pandemic; epidemiology.

INTRODUCTION

During the COVID-19 pandemic, non-pharmaceutical interventions have been implemented to reduce and control transmission of SARS-CoV-2 [1, 2]. As a result, several changes to the health system have had a significant impact on healthcare. Many outpatient services were limited or suspended to prevent the spread of the virus and to conserve resources for COVID-19 care [3–5]. As a result, routine check-ups, elective procedures and some specialist services have been postponed or canceled. Many healthcare providers turned to telemedicine to continue providing care while minimizing face-to-face contact. This allowed consultations, follow-ups and even some diagnostics to be carried out remotely [6]. However, it also highlighted inequalities in access to technology and reliable internet. Laboratories had to focus on COVID-19 testing, resulting in reduced availability of other diagnostic tests. Many tests and services that were not related to COVID-19 were de-prioritized, which had an impact on the monitoring and management of chronic diseases and other health issues [7]. Clinics and hospitals have restructured their physical spaces to improve their infection control measures. This included creating separate areas for COVID-19 patients, reconfiguring waiting rooms to promote social distancing, and implementing stricter hygiene protocols. Fear of COVID-19 infection also led many patients to delay or avoid medical care altogether [6,8].

The disruption to health services caused by the COVID-19 pandemic has had a notable and worrying impact on other areas of public health, particularly in the area of HIV testing and diagnosis [7, 9, 10]. In several countries, there has been a significant decline in HIV testing as a result of the diversion of health care resources to the COVID-19 response [11, 12]. In Belgium, there was a 50% decrease in HIV tests [13]. In Australia, testing decreased by 31% in a large STI clinic [14]. In China, a 59% reduction in testing across four regions [15]. In Kenya, challenges included a reduction in clinic-based testing and limited distribution of self-testing kits [16]. A study in Europe reported a more than 50% drop in HIV testing from March to May 2020, with a slight recovery in the following months [17]. In Japan, a similar decline in HIV testing has been observed [18]. To our knowledge, only two studies have assessed changes in HIV diagnoses in Peru [19, 20]. A study found that there was a decrease (33%) in the average number of HIV diagnoses in 2020 (n=5170), compared to the average number of cases between 2017 and 2019 (n=7746). This decrease in the average number of HIV diagnoses was observed in 21 out of 25 regions [19]. Although HIV occurs mainly in men aged 30 and over, the study did not analyze changes in HIV diagnosis by sex and age. It also did not analyze whether changes in HIV diagnoses were sustained in 2021 and 2022 (pandemic period).

Changes in HIV diagnosis have important implications for the timely initiation of treatment and linkage to care. In addition, undiagnosed and untreated HIV increases the risk of

transmitting to others, potentially exacerbating the epidemic. Therefore, knowing the impact of the COVID-19 pandemic on the detection of new HIV cases will help guide public health resources to reactivate HIV prevention and testing efforts. In addition, given the multiple "waves" of COVID-19 in Peru, a longer-term assessment of whether subsequent changes in restrictions have had an impact on HIV testing and whether or not testing rates are recovering is warranted.

To better understand how the implementation of COVID-19-related restrictive measures affected HIV infection care systems in Peru, we evaluated changes in HIV case reporting and the incidence rate in each of the COVID-19 pandemic years (2020, 2021 and 2022) compared with the pre-pandemic period (2015-19).

METHODS

Study design and data sources

We performed a retrospective cross-sectional study following the Strengthening the Reporting of Observational Studies in Epidemiology reporting guidelines (STROBE, S1 Table) [21]. In this observational study, we used HIV surveillance data from 1 January 2015 to 31 December 2022 from all 25 regions of Peru that are administrative departments. HIV surveillance data included official and valid information on new confirmed cases between 2015 and 2022, disaggregated by sex, age and region.

Data were collected from 25 geographic regions by the Epidemiological situation of HIV-AIDS in Peru, Centro Nacional de Epidemiología, Prevención y Control de Diseases, Ministerio de Salud del Peru (website: https://www.dge.gob.pe/vih/#grafico02) [22]. All new cases of HIV infection confirmed between 1 January 2015 and 31 December 2022 were included in the analysis. Data collected included the number of HIV cases by age group (0 to 11, 12 to 17, 18 to 29, 30 to 59 and ≥60 years), sex (male and female) and geographical region for each year. Other information not available on Centro Nacional de Epidemiología, Prevención y Control de Diseases website.

Outcome

The changes in the reporting of HIV cases (in terms of numbers and percentages) during the COVID-19 pandemic (in 2020, 2021 and 2022) were the primary outcome. The incidence of HIV infection before and during the COVID-19 pandemic was assessed as a secondary outcome.

Statistical analyses

We estimated the changes in HIV case reporting (in numbers and percentages) in the COVID-19 pandemic years (2020, 2021 and 2022), by sex and age, and in each geographical region. The average number of HIV cases in the years 2015-2019 was used to calculate the number of HIV cases in the pre-pandemic period. This average number of HIV cases by gender, age and region in the 5 years before the pandemic was the reference number for the comparison with the pandemic years of COVID-19 (2020, 2021 and 2022). Changes in HIV case notification during the pandemic period were estimated as the difference between HIV cases observed in 2020, 2021 and 2022 and the average number of HIV cases reported between 2015 and 2019. Changes in the proportion of HIV cases reported during the pandemic were calculated as: ((number of HIV cases reported in 2020, 2021 or 2022 - average number of HIV cases in the years 2015-2019)/average number of HIV cases in the years 2015-2019 × 100%) by sex, age and region. The yearly crude incidence rate (IR) for HIV per 100 000 inhabitants was calculated as the number of HIV cases divided by the yearly population estimates (by sex, age or region) and multiplied by 100 000. Exact 95% CIs were calculated for all IRs. Age-, sex- and region-specific incidence rates between 2015 and 2019 were calculated as the total number of HIV cases during the period divided by the sum of the annual population estimates for the 5 years, expressed per 100 000 inhabitants (by sex, age or region). IR ratios (IRRs) for HIV cases per sex, age and region were calculated to compare annual incidence rates during the COVID-19 pandemic period (2020, 2021 and 2022) and compared with the pooled pre-pandemic incidence. 95% CIs for the IRRs were calculated. The population figures used for the calculation of the IR were obtained from the projections of the National Institute of Statistics and Informatics of Peru. We created graphs to present the rates of HIV cases, including possible changes.

Ethics

This study was approved by the Institutional Review Board of Universidad Tecnologica del Peru (Lima, Peru). Informed consent was not required due to the retrospective, observational nature of the study.

RESULTS

From 1 January 2015 to 31 December 2022, there were 65 166 new cases of HIV infection. In the first year of the COVID-19 pandemic, there was a decrease (26%) in the number of HIV diagnoses (n = 6025), compared with the average number of cases between 2015 and 2019 (n = 8141). In the second and third years of the COVID-19 pandemic, there was an increased number of HIV diagnoses (6.7% and 19.8%, respectively), compared with the average number of cases between 2015 and 2019 (**Table 1**). In addition, in the first year of the

COVID-19 pandemic, the number of HIV diagnoses decreased in 21 out of 25 regions compared with the average number of cases between 2015 and 2019. In the second and third years of the pandemic, the number of HIV diagnoses decreased in 7 and 5 regions, respectively, compared with the average number of cases between 2015 and 2019 (**Table 1**).

Table 1. HIV cases before and during the COVID-19 pandemic in Peru, 2020-2022

	Pre-pandemic (2015–19)	2020			2021			2022		
	Mean	n	Differences in the number of cases	(%)	n	Differences in the number of cases	(%)	n	Differences in the number of cases	(%)
**Total**	8141	6025	-2116	-26.0	8684	543	6.7	9750	1609	19.8
**Sex**										
Male	6336	4650	-1686	-26.6	6997	661	10.4	7808	1472	23.2
Female	1805	1375	-430	-23.8	1687	-118	-6.5	1942	137	7.6
**Age group (years)**										
0 – 11	94	36	-58	-61.6	44	-50	-53.1	68	-26	-27.5
12 – 17	162	128	-34	-21.2	191	29	17.6	298	136	83.5
18 – 29	3659	2818	-841	-23.0	4008	349	9.5	4364	705	19.3
30 – 59	3960	2862	-1098	-27.7	4184	224	5.7	4716	756	19.1
≥60	265	181	-84	-31.7	257	-8	-3.1	304	39	14.6
**Age group in men (years)**										
0 – 11	54	20	-34	-63.0	25	-29	-53.7	42	-12	-22.2
12 – 17	96	67	-29	-29.9	111	15	16.1	178	82	86.2
18 – 29	2942	2225	-717	-24.4	3323	381	13.0	3632	690	23.5
30 – 59	3038	2200	-838	-27.6	3338	300	9.9	3715	677	22.3
≥60	206	138	-68	-33.0	200	-6	-2.9	241	35	17.0
**Age group in women (years)**										
0 – 11	40	16	-24	-59.8	19	-21	-52.3	26	-14	-34.7
12 – 17	67	61	-6	-8.7	80	13	19.8	120	53	79.6
18 – 29	717	593	-124	-17.3	685	-32	-4.5	732	15	2.1
30 – 59	922	662	-260	-28.2	846	-76	-8.2	1001	79	8.6
≥60	59	43	-16	-27.4	57	-2	-3.7	63	4	6.4
**Region**										
Amazonas	221	125	-96	-43.5	159	-62	-28.2	384	163	73.4
Ancash	137	87	-50	-36.7	174	37	26.6	196	59	42.6
Apurimac	10	13	3	32.7	25	15	155.1	29	19	195.9
Arequipa	324	153	-171	-52.7	232	-92	-28.3	254	-70	-21.5
Ayacucho	51	48	-3	-6.3	61	10	19.1	56	5	9.4
Cajamarca	62	50	-12	-18.8	99	37	60.7	111	49	80.2
Callao	706	407	-299	-42.4	449	-257	-36.4	451	-255	-36.2
Cusco	120	140	20	16.9	198	78	65.3	220	100	83.6
Huancavelica	17	11	-6	-33.7	12	-5	-27.7	14	-3	-15.7
Huanuco	93	60	-33	-35.8	64	-29	-31.5	99	6	6.0
Ica	237	152	-85	-35.8	241	4	1.9	234	-3	-1.1
Junin	161	139	-22	-13.7	204	43	26.7	190	29	18.0
La Libertad	415	284	-131	-31.6	459	44	10.6	539	124	29.9
Lambayeque	254	211	-43	-17.0	380	126	49.5	354	100	39.3
Lima	3614	2721	-893	-24.7	3904	290	8.0	4282	668	18.5
Loreto	569	388	-181	-31.9	614	45	7.8	699	130	22.8
Madre de Dios	78	72	-6	-7.7	82	4	5.1	85	7	9.0
Moquegua	39	30	-9	-23.9	43	4	9.1	48	9	21.8
Pasco	8	14	6	70.7	15	7	82.9	23	15	180.5
Piura	257	252	-5	-1.8	357	100	39.1	421	164	64.1

	Pre-pandemic (2015–19)	2020			2021			2022		
	**Mean**	**n**	**Differences in the number of cases**	**(%)**	**n**	**Differences in the number of cases**	**(%)**	**n**	**Differences in the number of cases**	**(%)**
Puno	64	62	-2	-3.1	104	40	62.5	115	51	79.7
San martin	194	173	-21	-10.6	230	36	18.8	270	76	39.5
Tacna	102	65	-37	-36.0	76	-26	-25.2	100	-2	-1.6
Tumbes	69	77	8	11.0	62	-7	-10.7	126	57	81.6
Ucayali	337	289	-48	-14.2	435	98	29.1	450	113	33.5

Compared with the pre-pandemic period overall incidence of 25.03 (95% CI, 24.48–25.58) per 100 000 habitants, the HIV overall incidence was 26% lower in 2020 (IRR=0.74; 95% CI, 0.71–0.76) and 5% higher in 2021 (IRR=1.05; 95% CI, 1.02–1.08). By 2022, HIV overall incidence was higher to the pre-pandemic period (IR=29.19; 95% CI, 28.61–29.78; **Table 2**).

Table 2. HIV incidence by sex and age before and during COVID-19 pandemic in Peru, 2020–2022

	Pre-pandemic (2015-19)		2020			2021			2022		
	**n**	**IR (95% CI)**	**n**	**IR (95% CI)**	**IRR (95% CI)**	**n**	**IR (95% CI)**	**IRR (95% CI)**	**n**	**IR (95% CI)**	**IRR (95% CI)**
**Total**	8141	25.03 (24.48-25.58)	6025	18.47 (18.00-18.94)	0.74 (0.71-0.76)	8684	26.28 (25.73-26.84)	1.05 (1.02-1.08)	9750	29.19 (28.61-29.78)	1.16 (1.13-1.20)
**Sex**											
Male	6336	39.57 (38.60-40.55)	4516	27.89 (27.10-28.70)	0.71 (0.68-0.73)	6895	41.2 (40.24-42.19)	1.04 (1.01-1.08)	7218	43.63 (42.63-44.65)	1.10 (1.06-1.14)
Female	1805	10.93 (10.43-11.45)	1346	8.19 (7.76-8.64)	0.75 (0.69-0.80)	1635	10.0 (9.55-10.53)	0.92 (0.86-0.98)	1808	10.73 (10.20-11.20)	0.98 (0.92-1.05)
**Age (years)**											
0–11	94	1.38 (1.11-1.69)	35	0.54 (0.37-075)	0.39 (0.26-0.58)	43	0.7 (0.50-0.90)	0.48 (0.33-0.70)	80	1.25 (0.90-1.55)	0.90 (0.66-1.23)
12–17	162	4.65 (3.96-5.41)	130	4.19 (3.50-4.98)	0.90 (0.71-1.14)	190	6.1 (5.23-6.98)	1.30 (1.05-1.62)	295	9.41 (8.36-10.54)	2.03 (1.67-2.47)
18–29	3659	54.15 (52.41-55.93)	2758	42.94 (41.36-44.58)	0.79 (0.75-0.83)	3951	62.0 (60.07-63.95)	1.14 (1.09-1.20)	4026	63.67 (61.72-65.66)	1.18 (1.12-1.23)
30–59	3960	33.01 (31.99-34.05)	2765	22.14 (21.32-22.98)	0.67 (0.64-0.70)	4093	32.1 (31.12-33.09)	0.97 (0.93-1.01)	4339	33.48 (32.49-34.49)	1.01 (0.97-1.06)
≥60	265	7.65 (6.75-8.62)	174	4.20 (3.60-4.90)	0.55 (0.45-0.67)	253	5.9 (5.20-6.66)	0.77 (0.64-0.92)	286	6.48 (5.75-7.28)	0.85 (0.71-1.01)
**Age in men (years)***											
0–11	54	1.18 (0.85-1.61)	19	0.58 (0.34-0.90)	0.48 (0.27-0.86)	26	0.8 (0.60-1.16)	0.67 (0.39-1.12)	51	1.57 (1.20-2.10)	1.33 (0.86-2.05)
12–17	96	6.40 (5.27-7.69)	66	4.23 (3.30-5.40)	0.66 (0.48-0.90)	110	7.1 (5.80-8.50)	1.10 (0.84-1.44)	174	11.1 (9.53-12.90)	1.74 (1.36-2.22)
18–29	2942	115.69 (112.06-119.42)	2171	70.43 (67.50-73.0)	0.61 (0.57-0.64)	3288	106.7 (103.06-110.40)	0.92 (0.88-0.97)	3339	109.7 (106.04-13.52)	0.94 (0.91-1.00)
30–59	3038	62.84 (60.82-64.91)	2125	33.78 (32.40-35.60)	0.54 (0.51-0.56)	3272	52.0 (50.30-53.83)	0.83 (0.79-0.86)	3426	52.5 (50.78-54.31)	0.84 (0.79-0.88)
≥60	206	14.83 (13.03-16.81)	135	6.84 (5.74-8.10)	0.46 (0.31-0.57)	199	10.1 (8.74-11.59)	0.68 (0.56-0.82)	228	10.9 (9.57-12.46)	0.74 (0.61-0.89)
**Age in women (years)***											
0–11	40	0.95 (0.65-1.35)	16	0.50 (0.29-0.82)	0.52 (0.26-0.98)	17	0.53 (0.30-0.80)	0.53 (0.28-0.99)	29	0.92 (0.60-1.32)	0.96 (0.56-1.63)
12–17	67	4.76 (3.78-5.90)	64	4.15 (3.20-5.30)	0.87 (0.61-1.22)	80	5.19 (4.12-6.46)	1.09 (0.79-1.50)	121	7.70 (6.39-9.20)	1.62 (1.21-2.17)
18–29	717	24.94 (23.30-26.66)	587	17.57 (16.20-19.10)	0.70 (0.63-0.78)	663	19.85 (18.37-21.42)	0.79 (0.72-0.88)	687	20.94 (19.40-22.57)	0.84 (0.75-0.93)
30–59	922	16.21 (15.22-17.24)	640	10.32 (9.54-11.15)	0.63 (0.57-0.70)	821	13.24 (12.35-14.18)	0.82 (0.74-0.90)	913	14.18 (13.28-15.13)	0.87 (0.80-0.96)

	Pre-pandemic (2015-19)		2020			2021			2022		
	n	IR (95% CI)	n	IR (95% CI)	IRR (95% CI)	n	IR (95% CI)	IRR (95% CI)	n	IR (95% CI)	IRR (95% CI)
≥60	59	4.02 (3.15-5.05)	39	1.80 (1.28-2.46)	0.45 (0.29-0.67)	54	2.49 (1.87-3.25)	0.62 (0.43-0.89)	58	2.49 (1.89-3.22)	0.62 (0.43-0.87)

Abbreviations: IR, incidence risk; IRR, incidence risk ratio; CI, confidence interval. *Only population for 2019, 2020, 2021 and 2022 was available.

Compared with the pre-pandemic period, the annual incidence of HIV among men decreased in 2020 (IR=27.89; 95% CI, 27.10–28.70) (**Table 2**). This decrease was 29% lower in 2020 (IRR = 0.71; 95% CI, 0.68–0.73), and 4% higher in 2021 (IRR=1.04, 95% CI, 1.01-1.08) and 10% higher in 2022 (IRR=1.10; 95% CI, 1.06–1.14) compared with the annual incidence of HIV among men during the pre-pandemic period (**Table 2**).

In the age-group stratified analysis, in comparison with the pre-pandemic period, the annual HIV incidence decreased in 2020 for all age groups, mainly for the 18–29 (IR=42.94; 95% CI, 41.36–44.58) and 30–59 (IR=22.14; 95% CI, 21.32–22.98). This decrease was 21 and 33% lower in 2020 for those aged 18–29 (IRR=0.79; 95% CI, 0.75–0.83) and 30–59 (IRR=0.67; 95% CI, 0.64–0.70), respectively, compared to the pre-pandemic period. There was also a higher incidence for the years 2021 and 2022. Similar trends were observed for annual HIV incidence in the stratified analysis by age group compared with the overall analysis in men, with decreases of 39% and 46% in 2020 for those aged 18–29 years (IRR=0.61; 95% CI, 0.57–0.64) and 30–59 years (IRR=0.54; 95% CI, 0.51–0.56), respectively (**Table 2**).

Compared with the pre-pandemic period, annual HIV incidence has decreased in 11 out of 25 regions in 2020. In the regions of Amazonas, Callao, Loreto, Madre de Dios and Ucayali, where the annual HIV incidence was the highest in the pre-pandemic period, this incidence was 45% (IRR=0.55; 95% CI, 0.44–0.69), 47% (IRR=0.53; 95% CI, 0.47–0.60), 33% (IRR=0.67, 95% CI, 0.59–0.77), 19% (IRR=0.81; 95% CI, 0.5846–1.13) and 25% (IRR=0.75; 95% CI, 0.64–0.88) lower in 2020, respectively (**Table 3**). In Callao region, the annual incidence of HIV was also 43% lower in 2021 (IRR=0.57; 95% CI, 0.51–0.65) and 49% lower in 2022 (IRR=0.51; 95% CI, 0.45–0.58), compared with the pre-pandemic period. In Amazonas region, the annual incidence of HIV was 35% lower in 2021 (IRR=0.65; 95% CI, 0.52–080) increasing to more than 60% in 2022 (IRR=1.60.93; 95% CI, 1.35–1.90), compared with the pre-pandemic period. Finally, in Madre de Dios region, the annual incidence of HIV was 14% lower in 2021 (IRR=0.68; 95% CI, 0.62–1.19) and 15% lower in 2022 (IRR=0.85; 95% CI, 0.62–1.17), compared with the pre-pandemic period (**Table 3**).

Table 3. HIV incidence by region before and during COVID-19 pandemic in Peru, 2020–2022

	Pre-pandemic (2015-19)		2020			2021			2022		
**Region**	**n**	**IR (95% CI)**	**n**	**IR (95% CI)**	**IRR (95% CI)**	**n**	**IR (95% CI)**	**IRR (95% CI)**	**n**	**IR (95% CI)**	**IRR (95% CI)**
Amazonas	221	51.85 (45.15-59.04)	122	28.58 (23.70-34.10)	0.55 (0.44-0.69)	145	33.42 (28.20-39.30)	0.65 (0.52-0.80)	363	82.86 (74.60-91.80)	1.60 (1.35-1.90)
Ancash	137	11.73 (9.82-13.82)	89	7.54 (6.10-9.30)	0.64 (0.49-0.85)	175	14.88 (12.80-17.30)	1.27 (1.01-1.60)	196	16.60 (14.40-19.10)	1.42 (1.13-1.78)
Apurimac	10	2.10 (1.03-3.94)	10	2.32 (1.10-4.30)	1.08 (0.40-2.90)	25	5.89 (2.80-8.70)	2.75 (1.28-6.42)	27	6.38 (4.20-9.30)	2.98 (1.40-6.90)
Arequipa	324	23.96 (21.45-26.75)	150	10.02 (8.50-11.70)	0.42 (0.34-0.51)	233	15.27 (22.40-17.40)	0.64 (0.53-0.76)	237	15.39 (13.50-17.50)	0.64 (0.54-0.76)
Ayacucho	51	7.12 (5.28-9.33)	48	7.18 (5.30-9.50)	1.01 (0.67-1.53)	62	9.42 (7.20-12.10)	1.34 (0.90-1.96)	57	8.65 (6.50-11.20)	0.93 (0.64-1.34)
Cajamarca	62	3.99 (3.08-5.15)	51	3.51 (2.60-4.60)	0.87 (0.59-1.28)	99	6.84 (4.60-8.30)	1.70 (1.23-2.38)	104	7.19 (5.90-8.70)	1.79 (1.29-2.49)
Callao	706	66.15 (61.33-71.18)	396	35.05 (31.70-38.70)	0.53 (0.47-0.60)	434	37.82 (61.40-41.60)	0.57 (0.51-0.65)	394	34.02 (30.70-37.60)	0.51 (0.45-0.58)
Cusco	120	8.90 (7.39-10.66)	139	10.24 (8.60-12.10)	1.15 (0.89-1.48)	196	14.41 (11.50-16.00)	1.61 (1.28-2.05)	217	15.90 (13.90-18.20)	1.78 (1.42-2.25)
Huancavelica	17	3.26 (1.95-5.35)	11	3.01 (1.50-5.40)	0.90 (0.38-2.04)	13	3.68 (2.00-6.30)	1.10 (0.49-2.41)	14	4.02 (2.20-6.70)	1.20 (0.55-2.59)
Huanuco	93	10.50 (8.44-12.81)	57	7.50 (5.70-9.70)	0.72 (0.51-1.01)	58	7.66 (9.80-9.90)	0.73 (0.52-1.03)	96	12.74 (10.30-15.60)	1.22 (0.91-1.64)
Ica	237	28.93 (25.4-32.91)	147	15.07 (12.70-17.70)	0.52 (0.42-0.64)	219	22.27 (24.40-25.40)	0.77 (0.64-0.93)	201	20.09 (17.40-23.10)	0.69 (0.57-0.84)
Junin	161	11.58 (9.86-13.52)	137	10.06 (8.50-11.90)	0.87 (0.69-1.09)	203	14.96 (13.90-17.20)	1.29 (1.04-1.59)	184	13.53 (11.60-15.60)	1.17 (0.94-1.45)
La Libertad	415	21.21 (19.22-23.35)	280	13.88 (12.30-15.60)	0.65 (0.56-0.76)	449	22.10 (23.10-24.20)	1.04 (0.91-1.19)	495	24.10 (22.00-26.30)	1.14 (0.99-1.30)
Lambayeque	254	19.54 (17.20-22.08)	195	14.88 (12.90-17.10)	0.76 (0.63-0.92)	372	28.02 (15.30-31.00)	1.43 (1.22-1.69)	342	25.56 (22.90-28.40)	1.31 (1.11-1.54)
Lima	3614	34.55 (33.44-35.70)	2634	24.78 (23.80-25.70)	0.72 (0.68-0.75)	3835	35.16 (41.10-36.30)	1.02 (0.97-1.06)	3829	34.82 (33.70-35.90)	1.01 (0.96-1.05)
Loreto	569	52.83 (48.54-57.31)	366	35.62 (32.10-39.50)	0.67 (0.59-0.77)	597	57.32 (63.80-62.10)	1.08 (0.97-1.22)	668	63.86 (59.10-68.90)	0.93 (0.83-1.03)
Madre de dios	78	51.94 (41.05-64.82)	73	42.00 (32.90-52.80)	0.81 (0.58-1.13)	82	44.77 (53.60-55.60)	0.86 (0.62-1.19)	83	44.13 (35.10-54.70)	0.85 (0.62-1.17)
Moquegua	39	20.96 (14.76-28.37)	30	15.57 (10.50-22.0)	0.75 (0.45-1.24)	42	21.58 (18.50-29.20)	1.04 (0.66-1.65)	48	24.64 (18.20-32.70)	1.19 (0.76-1.86)
Pasco	8	2.62 (1.10-5.04)	14	5.15 (2.81-8.64)	2.01 (0.79-5.54)	15	5.49 (3.07-9.06)	2.15 (0.85 - 5.85)	23	8.54 (5.41-12.81)	3.34 (1.44-8.63)
Piura	257	13.49 (11.91-15.27)	249	12.16 (10.70-13.80)	0.90 (0.75-1.07)	353	16.93 (20.20-18.80)	1.25 (1.06-1.48)	419	19.87 (18.00-21.90)	1.47 (1.26-1.72)
Puno	64	4.35 (3.35-5.55)	61	4.93 (3.80-6.30)	1.13 (0.78-1.63)	97	7.98 (4.50-9.70)	1.57 (1.15-2.15)	113	9.42 (7.80-11.30)	2.16 (1.58-2.99)
San Martin	194	21.88 (18.95-25.24)	158	17.56 (14.90-20.50)	0.80 (0.64-0.99	220	24.26 (23.20-27.70)	1.84 (1.33-2.56)	251	27.35 (24.10-31.00)	1.24 (1.03-1.51)
Tacna	102	28.36 (23.21-34.56)	64	17.25 (13.30-22.00)	0.61 (0.44-0.84)	76	20.09 (33.80-25.10)	0.71 (0.52-0.96)	99	25.93 (21.10-31.60)	0.91 (0.68-1.21)
Tumbes	69	27.89 (21.57-35.09)	78	31.01 (24.50-38.70)	1.12 (0.80-1.57)	60	23.40 (33.90-30.10)	0.84 (0.59-1.21)	125	48.16 (40.10-57.40)	1.74 (1.28-2.36)
Ucayali	337	65.03 (58.28-72.36)	289	49.06 (43.60-55.00)	0.75 (0.64-0.88)	434	71.61 (62.00-78.70)	1.10 (0.95-1.27)	443	71.96 (65.40-78.90)	1.11 (0.96-1.28)

Abbreviations: IR, incidence risk; IRR, incidence risk ratio; CI, confidence interval; NA, no applicable.

DISCUSSION

Our findings shown a reduction in the incidence of HIV infection (26% reduction) in 2020 compared with the pre-pandemic period in Peruvian population. Although limited, some studies have reported a decrease in HIV incidence during the COVID-19 pandemic in 2020 [23, 24]. During the COVID-19 pandemic, many outpatient services were limited or suspended to

prevent the spread of SARS-CoV-2 and to conserve resources for COVID-19 care [3-5], including in Peru [25]. Consequently, laboratories had to focus on COVID-19 testing, resulting in a reduction in the availability of other diagnostic tests, particularly in the area of HIV testing and diagnosis (with reductions of up to 50% in HIV testing in some countries) [7, 9, 10]. In Peru, the average number of HIV diagnoses decreased by 33% by 2020 [19]. According to a recent study, the proportion of the Peruvian population tested for HIV in the past year decreased by an average of 8.33% (95% CI: -10.73% to -5.93%) [20]. Thus, our findings of reduced HIV incidence are consistent with the reduction in the proportion of HIV tests conducted by 2020. In addition, our results showed a temporal association between the implementation of non-pharmaceutical interventions to reduce the spread of SARS-CoV-2 and the incidence of HIV infection in 2020. In response to the decline in the HIV infection rate, the Ministry of Health of Peru has implemented several strategies to improve HIV testing and treatment services, particularly at the primary health care level. These measures include the decentralization of molecular testing, the introduction of contact tracing systems, and the decentralization of antiretroviral treatment [29]. We also observed that the incidence of HIV infection began to return to pre-pandemic rates in 2021 (with an increase of 5%), which was temporally associated with reducing or eliminating non-pharmaceutical interventions. This finding also correlates with the decrease in the Peruvian population tested for HIV in 2021 [20]. Some studies also showed that HIV screening declines by 2021 during the second or third COVID-19 pandemic wave [26, 27]. A modeled study also found that the largest estimated impact on HIV would come from interruptions in antiretroviral therapy, which could occur during a period of high demand on the health system [28]. By 2022, the incidence of HIV infection was higher to that during the pre-pandemic period. According to the literature, the Peruvian health system achieved partial or complete recovery and was able to care for several patients in 2022, as compared to before the pandemic [25].

Although HIV incidence decreased in both sexes, the annual incidence of HIV among men was 29% lower in 2020, compared with the pre-pandemic period, mainly for the 18–29 and 30–59 age groups. This change could be influenced by several factors. First, the pandemic caused a shift in healthcare priorities, with resources being reallocated to COVID-19 efforts. This likely led to fewer HIV tests being conducted and, consequently, fewer diagnoses being recorded in men [20], who are the population most affected by HIV infection. Second, lockdowns, social distancing measures, and restrictions on gatherings may have reduced opportunities for sexual activity with new partners, potentially decreasing HIV transmission rates. Third, access to HIV prevention services, such as pre-exposure prophylaxis (PrEP), condom distribution, and education programs, may have been disrupted. Data from some studies show that PrEP use has declined during the pandemic, likely resulting in fewer clinic-based

PrEP follow-up visits [30, 31]. This may have affected the trends observed, as reported in some countries [31–33]. Fourth, the economic and social disruptions of the pandemic could have altered behavior, including changes in migration patterns or access to healthcare, influencing HIV transmission dynamics. Compared with the pre-pandemic period, in 2021 (4% higher) and 2022 (10% higher) the annual incidence of HIV among men has increased. The observed decline in HIV incidence during this period should be interpreted with caution. It is likely that the reduced incidence reflects temporary changes due to the pandemic.

In 2020, 11 out of 25 Peruvian regions experienced a decline in annual HIV incidence compared to the pre-pandemic period. This decrease was most pronounced in regions with the highest pre-pandemic incidence rates, including Amazonas, Callao, Loreto, Madre de Dios, and Ucayali. Although there was a decreased in incidence of HIV infection, it is highly likely that HIV continues to spread in communities. HIV undiagnosis remains a significant public health problem in Peru; studies indicate that a significant number of people living with HIV are unaware of their status, challenging epidemic control, especially among at-risk groups [34-36]. As a concentrated epidemic, HIV is most prevalent in men who have sex with men, transgender women, and sex workers. Prior to the pandemic, a study of 378 men who have sex with men and transgender women in the Lima region found that 31% were infected with HIV, of whom 35% had not been previously diagnosed [35]. Another study conducted in the Cajamarca region between 2015 and 2021 estimated that of 1,388 people living with HIV, approximately 393 cases (28.4%) were undiagnosed [36]. Another group considered vulnerable are indigenous peoples, specifically from the Amazon regions of Peru such as Amazonas, Loreto and Ucayali. Preliminary evidence suggests that the conception and social representations of health and illness, especially in the case of HIV infection in indigenous populations, strongly influence the behaviors that they ultimately exhibit in response to health interventions [37,38]. Studies indicate that a significant number of people living with HIV are unaware of their status, which poses a challenge to achieving epidemic control. Although the COVID-19 pandemic had receded by 2021 and 2022, the burden of HIV infection had increased in Peruvian regions with high prevalence of HIV infection. To address this problem, public health initiatives have been undertaken. The Centers for Disease Control and Prevention (CDC) and Peruvian health authorities have focused on outreach testing for key populations, index testing services (testing partners of HIV-infected persons), provider-initiated testing, and counseling. These approaches aim to increase diagnosed and reduce undiagnosed cases.

Limitations

Our study has several limitations. First, our study was not able to address whether changes in health-seeking behavior, including a reluctance to visit health facilities (especially in

2020), may have contributed to the lower incidence of HIV infection during the COVID-19 pandemic. Second, only population data for the years 2019, 2020, 2021, and 2022 were available to estimate incidence by age group among males and females. Therefore, the lack of population data from 2015 to 2018 may have biased the estimates of expected incidence by age group by sex. Strengths of our study include five years of pre-pandemic surveillance, whereas similar surveillance data are scarce elsewhere in Latin America. As a result, our study provides the first and probably most comprehensive insights into the impact of the COVID-19 pandemic on HIV incidence rates and the burden of disease during the three years of the COVID-19 pandemic.

Conclusions

Our study shows that during the COVID-19 pandemic in 2020, there was a decrease in the incidence of HIV infection in the Peruvian population. However, this incidence started to return to pre-pandemic rates as the COVID-19 pandemic receded, which coincided with the reduction or elimination of non-pharmaceutical interventions. By 2022, the incidence of HIV infection was higher to pre-pandemic levels, especially in regions of the Peruvian Amazon. In addition, the overall impact of reduced incidence could lead to delayed initiation of antiretroviral treatment, poor viral suppression, increased risk of transmission and ultimately an increase in HIV-related deaths.

REFERENCES

1. Liu Y, Morgenstern C, Kelly J, Lowe R; CMMID COVID-19 Working Group; Jit M. The impact of non-pharmaceutical interventions on SARS-CoV-2 transmission across 130 countries and territories. BMC Med. 2021 Feb 5;19(1):40. doi: 10.1186/s12916-020-01872-8.
2. He X, Chen H, Zhu X, Gao W. Non-pharmaceutical interventions in containing COVID-19 pandemic after the roll-out of coronavirus vaccines: a systematic review. BMC Public Health. 2024 Jun 6;24(1):1524. doi: 10.1186/s12889-024-18980-2.
3. Zachrison KS, Yan Z, Schwamm LH. Changes in Virtual and In-Person Health Care Utilization in a Large Health System During the COVID-19 Pandemic. JAMA Netw Open. 2021 Oct 1;4(10):e2129973. doi: 10.1001/jamanetworkopen.2021.29973.
4. Kiarie H, Temmerman M, Nyamai M, Liku N, Thuo W, Oramisi V, et al. The COVID-19 pandemic and disruptions to essential health services in Kenya: a retrospective time-series analysis. Lancet Glob Health. 2022 Sep;10(9):e1257-e1267. doi: 10.1016/S2214-109X(22)00285-6.

5. Xiao H, Dai X, Wagenaar BH, Liu F, Augusto O, Guo Y, Unger JM. The impact of the COVID-19 pandemic on health services utilization in China: Time-series analyses for 2016-2020. Lancet Reg Health West Pac. 2021 Mar 24;9:100122. doi: 10.1016/j.lanwpc.2021.100122.
6. Moynihan R, Sanders S, Michaleff ZA, Scott AM, Clark J, To EJ, et al. Impact of COVID-19 pandemic on utilisation of healthcare services: a systematic review. BMJ Open. 2021 Mar 16;11(3):e045343. doi: 10.1136/bmjopen-2020-045343.
7. Arsenault C, Gage A, Kim MK, Kapoor NR, Akweongo P, Amponsah F, et al. COVID-19 and resilience of healthcare systems in ten countries. Nat Med. 2022 Jun;28(6):1314-1324. doi: 10.1038/s41591-022-01750-1.
8. Haleem A, Javaid M, Vaishya R. Effects of COVID-19 pandemic in daily life. Curr Med Res Pract. 2020 Mar-Apr;10(2):78-79. doi: 10.1016/j.cmrp.2020.03.011.
9. Shoptaw S, Goodman-Meza D, Landovitz RJ. Collective Call to Action for HIV/AIDS Community-Based Collaborative Science in the Era of COVID-19. AIDS Behav. 2020 Jul;24(7):2013-2016. doi: 10.1007/s10461-020-02860-y.
10. SeyedAlinaghi S, Mirzapour P, Pashaei Z, Afzalian A, Tantuoyir MM, Salmani R, et al. The impacts of COVID-19 pandemic on service delivery and treatment outcomes in people living with HIV: a systematic review. AIDS Res Ther. 2023 Jan 6;20(1):4. doi: 10.1186/s12981-022-00496-7.
11. Mude W, Mwenyango H, Preston R, O'Mullan C, Vaughan G, Jones G. HIV Testing Disruptions and Service Adaptations During the COVID-19 Pandemic: A Systematic Literature Review. AIDS Behav. 2024 Jan;28(1):186-200. doi: 10.1007/s10461-023-04139-4.
12. Moitra E, Tao J, Olsen J, Shearer RD, Wood BR, Busch AM, et al. Impact of the COVID-19 pandemic on HIV testing rates across four geographically diverse urban centres in the United States: An observational study. Lancet Reg Health Am. 2022 Mar;7:100159. doi: 10.1016/j.lana.2021.100159.
13. Darcis G, Vaira D, Moutschen M. Impact of coronavirus pandemic and containment measures on HIV diagnosis. Epidemiol Infect. 2020 Aug 24;148:e185. doi: 10.1017/S0950268820001867.
14. Chow EPF, Ong JJ, Denham I, Fairley CK. HIV Testing and Diagnoses During the COVID-19 Pandemic in Melbourne, Australia. J Acquir Immune Defic Syndr. 2021 Apr 1;86(4):e114-e115. doi: 10.1097/QAI.0000000000002604.
15. Booton RD, Fu G, MacGregor L, Li J, Ong JJ, Tucker JD, et al. The impact of disruptions due to COVID-19 on HIV transmission and control among men who have sex with men in China. J Int AIDS Soc. 2021 Apr;24(4):e25697. doi: 10.1002/jia2.25697.

16. Kimanga DO, Makory VNB, Hassan AS, Ngari F, Ndisha MM, Muthoka KJ, et al. Impact of the COVID-19 pandemic on routine HIV care and antiretroviral treatment outcomes in Kenya: A nationally representative analysis. PLoS One. 2023 Nov 27;18(11):e0291479. doi: 10.1371/journal.pone.0291479.
17. Simões D, Stengaard AR, Combs L, Raben D; EuroTEST COVID-19 impact assessment consortium of partners. Impact of the COVID-19 pandemic on testing services for HIV, viral hepatitis and sexually transmitted infections in the WHO European Region, March to August 2020. Euro Surveill. 2020 Nov;25(47):2001943. doi: 10.2807/1560-7917.ES.2020.25.47.2001943.
18. Ejima K, Koizumi Y, Yamamoto N, Rosenberg M, Ludema C, Bento AI, et al. HIV Testing by Public Health Centers and Municipalities and New HIV Cases During the COVID-19 Pandemic in Japan. J Acquir Immune Defic Syndr. 2021 Jun 1;87(2):e182-e187. doi: 10.1097/QAI.0000000000002660.
19. Paredes JL, Owen R, Mejia F. Changes in the notification of HIV diagnoses during the COVID-19 pandemic in Peru. Sex Transm Infect. 2022 Nov;98(7):544. doi: 10.1136/sextrans-2022-055465.
20. Yrene-Cubas RA, Perez-Castilla J, Reynaga-Cottle DE, Bringas MJ, Soriano-Moreno DR, Fernandez-Guzman D, et al. The impact of the COVID-19 pandemic on HIV testing in Peru: an interrupted time series analysis from 2014 to 2022. BMC Infect Dis. 2025 Jan 7;25(1):39. doi: 10.1186/s12879-024-10407-y.
21. Vandenbroucke JP, von Elm E, Altman DG, Gøtzsche PC, Mulrow CD, Pocock SJ, Poole C, Schlesselman JJ, Egger M; STROBE Initiative. Strengthening the Reporting of Observational Studies in Epidemiology (STROBE): explanation and elaboration. PLoS Med. 2007 Oct 16;4(10):e297. doi: 10.1371/journal.pmed.0040297.
22. Center for Diseases Control (CDC) Epidemiological situation on HIV/AIDS in Peru, Ministry of Health, Peru. CDC-Peru: Lima; 2022.
23. Nishiura H, Fujiwara S, Imamura A, Shirasaka T. HIV incidence before and during the COVID-19 pandemic in Japan. Math Biosci Eng. 2024 Feb 29;21(4):4874-4885. doi: 10.3934/mbe.2024215.
24. Mitchell KM, Dimitrov D, Silhol R, Geidelberg L, Moore M, Liu A, et al. The potential effect of COVID-19-related disruptions on HIV incidence and HIV-related mortality among men who have sex with men in the USA: a modelling study. Lancet HIV. 2021 Apr;8(4):e206-e215. doi: 10.1016/S2352-3018(21)00022-9.
25. Villarreal-Zegarra D, Bellido-Boza L, Erazo A, Pariona-Cárdenas M, Valdivia-Miranda P. Impact of the COVID-19 pandemic on the services provided by the Peruvian health system: an analysis of people with chronic diseases. Sci Rep. 2024 Feb 13;14(1):3664. doi: 10.1038/s41598-024-54275-7.

26. Romero-Hernández B, Martínez-García L, Rodríguez-Dominguez M, Martínez-Sanz J, Vélez-Díaz-Pallarés M, Pérez Mies B, et al. The Negative Impact of COVID-19 in HCV, HIV, and HPV Surveillance Programs During the Different Pandemic Waves. Front Public Health. 2022;10:880435. doi: 10.3389/fpubh.2022.880435.
27. McFall AM, Menezes NP, Srikrishnan AK, Solomon SS, Anand S, Baishya JJ, et al. Impact of the COVID-19 pandemic on HIV prevention and care services among key populations across 15 cities in India: a longitudinal assessment of clinic-based data. J Int AIDS Soc. 2022 Jul;25(7):e25960. doi: 10.1002/jia2.25960.
28. Hogan AB, Jewell BL, Sherrard-Smith E, Vesga JF, Watson OJ, Whittaker C, et al. Potential impact of the COVID-19 pandemic on HIV, tuberculosis, and malaria in low-income and middle-income countries: a modelling study. Lancet Glob Health. 2020 Sep;8(9):e1132-e1141. doi: 10.1016/S2214-109X(20)30288-6.
29. Dirección de Prevención y Control de VIH-SIDA. Enfermedades de Transmisión Sexual y Hepatitis (DPVIH) - Contenido institucional - Ministerio de Salud - Plataforma del Estado Peruano. https://www.gob.pe/23137-ministerio-de-salud-direccion-de-prevencion-y-control-de-vih-sida-enfermedades-de-transmision-sexual-y-hepatitis-dpvih
30. Hammoud M.A., Grulich A., Holt M., et al. Substantial decline in use of HIV preexposure prophylaxis following introduction of COVID-19 physical distancing restrictions in Australia: results from a prospective observational study of gay and bisexual men. Jaids J Acquir Immune Defic. 2021;86(1):22–30. doi: 10.1097/QAI.0000000000002514.
31. Chow EPF, Hocking JS, Ong JJ, Schmidt T, Buchanan A, Rodriguez E, et al. Changing the Use of HIV Pre-exposure Prophylaxis Among Men Who Have Sex With Men During the COVID-19 Pandemic in Melbourne, Australia. Open Forum Infect Dis. 2020 Jul 2;7(7):ofaa275. doi: 10.1093/ofid/ofaa275.
32. Goedel WC, Rogers BG, Li Y, Nunn AS, Patel RR, Marshall BDL, et al. Pre-exposure Prophylaxis Discontinuation During the COVID-19 Pandemic Among Men Who Have Sex With Men in a Multisite Clinical Cohort in the United States. J Acquir Immune Defic Syndr. 2022 Oct 1;91(2):151-156. doi: 10.1097/QAI.0000000000003042.
33. Rogers BG, Tao J, Darveau SC, Maynard M, Almonte A, Napoleon S, et al. The Impact of COVID-19 on Sexual Behavior and Psychosocial Functioning in a Clinical Sample of Men who have Sex with Men Using HIV Pre-exposure Prophylaxis. AIDS Behav. 2022 Jan;26(1):69-75. doi: 10.1007/s10461-021-03334-5.
34. Vagenas P, Ludford KT, Gonzales P, Peinado J, Cabezas C, Gonzales F, et al. Being unaware of being HIV-infected is associated with alcohol use disorders and high-risk

sexual behaviors among men who have sex with men in Peru. AIDS Behav. 2014 Jan;18(1):120-7. doi: 10.1007/s10461-013-0504-2.

35. Billings JD, Joseph Davey DL, Konda KA, Bristow CC, Chow J, Klausner JD, et al. Factors associated with previously undiagnosed human immunodeficiency virus infection in a population of men who have sex with men and male-to-female transgender women in Lima, Peru. Medicine (Baltimore). 2016;95(42):e5147.
36. Quiroz-Ruiz HR, Pairazamán-Quiroz OD, Quiroz-Villanueva DE, Cornejo-Pacherres HD, Hernández-Palomino FN, Cruzado-Montero AA, et al. [Estimate of undiagnosed HIV cases in the Cajamarca region of Peru: probabilistic linkage between databases]. Cien Saude Colet. 2023;28(6):1843-1852. Spanish. doi: 10.1590/1413-81232023286.11922022.
37. Huamán B, Gushiken A, Benites C, Quiroz F, García-Fernández L. Prevención de la transmisión materno-infantil del VIH en gestantes y madres awajún y wampis de la región amazonas en Perú. Rev Peru Med Exp Salud Publica. 2017;34(4):627–32. doi: 10.17843/rpmesp.2017.344.2725.
38. Valenzuela-Oré F, Angulo-Bazán Y, Lazóriga-Sandoval LD, Cruz-Vilcarromero NL, Cubas-Sagardia CR. Factors influencing adherence to anti-retroviral therapy in amazonian indigenous people living with HIV/AIDS. BMC Public Health. 2023. 15;23(1):497. doi: 10.1186/s12889-023-15362-y.

Supporting information

S1 Table. STROBE Statement—Checklist of items that should be included in reports of cross-sectional studies

Click here to access/download
Supporting Information
Table S1. Checklist STROBE.doc
