# Supplementary material for: Changes in HIV incidence during the COVID-19 pandemic (2020–22) compared with the pre-pandemic period (2015–19) in Peru: An observational study
Source: PLoS One. 2025 Jun 2;20(6):e0324784. doi: 10.1371/journal.pone.0324784 (PMC12129149; doi:10.1371/journal.pone.0324784)
Supplement: Attachment — Submitted filename: Review.pdf [file pone.0324784.s005.pdf]

Thank you for the opportunity to review your manuscript. I appreciate the effort and the valuable insights presented. However, I have a few points I would like to comment on. In addition to the detailed revisions, I would like to highlight three general observations:

- English Writing Support: I strongly suggest that the authors seek assistance with the revision of the English writing. While the overall content is clear, certain sections would benefit from improved language flow and grammar to ensure clarity and ease of reading for a broader audience.

- Background of the Study: The background of the study could be expanded. It would be helpful to include more details about the condition being studied, how it is typically diagnosed, and how the COVID-19 pandemic may have affected diagnosis rates. Furthermore, discussing the implications of missed diagnoses during the pandemic would strengthen the background and provide more context for the study.

- Clarity and Systematization of Results: The results section could be discussed in a more systematic and clearer manner. Currently, the presentation of the results feels somewhat fragmented. A more structured approach would enhance the readability and help the readers better understand the findings and their implications.

Comments:

ABSTRACT

LINES 26-29

There are a couple of issues in this sentence that need correction. First, the phrase "from 25 geographically diverse from Peru" is unclear and likely missing a noun after "diverse". A suggested revision is "from 25 geographically diverse regions in Peru". Second, there is a subject-verb agreement error in "HIV incidence during the COVID-19 pandemic years (2020, 2021, and 2022) were compared". Since HIV incidence is singular, the verb should also be singular: "was compared". Please revise accordingly.

LINE 33

The words “By 2022” seem out of place.

LINE 35

Consider removing the “and” before 4% for smoother phrasing.

LINE 36

Age stratified should be age-stratified.

LINE 42

“Was” should be “has” to reflect the appropriate tense.

LINES 44-45

The sentence is grammatically incorrect. It should be: “By 2022, the incidence of HIV infection was higher than in the pre-pandemic period, especially in regions of the Peruvian Amazon."

<u>INTRODUCTION</u>

LINE 51
The authors mention non-pharmaceutical interventions but never explain what they are. Although this is not the main focus of the work, I think providing a brief explanation would improve the flow of the text.

LINE 73
The sentence “In China, a 59% reduction in testing across four regions” is incomplete and lacks a main verb. Consider revising it to something like, “In China, a 59% reduction in testing was observed across four regions.”

LINES 75-76
The phrase "a more than 50% drop" sounds slightly unnatural. A clearer construction would be "more than a 50% drop" or "a drop of more than 50%". Please consider revising for better readability.

LINES 77-78
The authors state that two studies assessed changes in HIV diagnoses in Peru but only present the results from one of them. Consider addressing why the second study's results are not presented or clarify the reasoning behind this.

LINES 84-85
I’m not sure if “changes” is the appropriate term for conveying that undiagnosed HIV cases have implications for treatment initiation. Consider using "delays" or "barriers" instead, if that is the intended meaning.

LINES 94-95
The correct form is “higher than the pre-pandemic period”, not “higher to the pre-pandemic period.”

<u>METHODS</u>

Study Design and Data Sources

LINE 98
The type of study is incorrect. A cross-sectional study does not have a temporal direction, so it is not correct to define a cross-sectional analysis as 'retrospective'.

LINES 110-111
I don’t understand what the authors mean with the sentence, “Other information not available on Centro Nacional de Epidemiología, Prevención y Control de Diseases website.” Consider clarifying what specific information was unavailable.

Statistical Analysis

Line 140
Authors state that they 'created graphs to present the rates of HIV cases, including possible changes,' but these were not provided.

Ethics

LINES 143
Please revise the concept of "retrospective" as it may be confusing, given that the study design appears to be cross-sectional.

RESULTS

LINES 167-170
The paragraph presents some inconsistencies in how the changes in HIV incidence are described, which could lead to confusion for the reader. Specifically, in the first sentence, you mention that the annual incidence of HIV among men decreased in 2020, but in the following sentences, the incidence rate ratios (IRR) for 2021 and 2022 show an increase in HIV incidence compared to the pre-pandemic period. For this reason, you cannot say, “this decrease was 4% higher…”Please revise for consistency.

LINES 171-175
In the sentence “This decrease was 21 and 33% lower in 2020 for those aged 18–29 (IRR=0.79; 95% CI, 0.75–0.83) and 30–59 (IRR=0.67; 95% CI, 0.64–0.70), respectively,” the phrase "lower" is not the most appropriate term to describe the decrease. Since the text is comparing the magnitude of the decrease in different age groups, it would be clearer to say “greater” instead of “lower” to convey that the decrease was more substantial in the 30–59 age group compared to the 18–29 age group. Please revise the entire manuscript accordingly.

DISCUSSION

LINES 200-201
Please explain what “although limited” means in this context. It’s unclear if this refers to the data, analysis, or the limitations of the study.

LINES 207-210
The authors state that HIV diagnoses reduced 33% in 2020, and then try to justify this finding with data that relates to the past year (2024?). I don’t believe that data with a 4 year difference could be correlated.

LINES 217-220
The authors state, “also observed that the incidence of HIV infection began to return to pre-pandemic rates in 2021 (with an increase of 5%), which was temporally associated with reducing or eliminating non-pharmaceutical interventions. This finding also correlates with the decrease in the Peruvian population tested for HIV in 2021.” I’m confused. How can an increase in HIV incidence be related to a decrease in testing? This seems contradictory to previous statements made by the authors. Could this section be revised for clarity?

LINES 222-224

The sentence “A modeled study also found that the largest estimated impact on HIV would come from interruptions in antiretroviral therapy, which could occur during a period of high demand on the health system” seems out of context, since the authors are talking about incidence rates and testing, and not about HIV therapy and its impact. Consider providing more context or removing this reference.

LINES 224-227

In the sentence “By 2022, the incidence of HIV infection was higher to that during the pre-pandemic period”, "higher to" is incorrect and should be substituted with "higher than". Additionally, the authors should contextualize and explain this result. The sentence “According to the literature, the Peruvian health system achieved partial or complete recovery and was able to care for several patients in 2022, as compared to before the pandemic” doesn’t justify the finding. Do the authors mean that the testing rate returned to pre-pandemic values? The only data related to this appear in lines 207-209, where the authors state that “... the proportion of the Peruvian population tested for HIV in the past year decreased by an average of 8.33% (95% CI: -10.73% to -5.93%)”. If this is the only data available, it doesn’t seem to explain the study's findings.

LINES 228-240

These arguments shouldn’t be presented to explain the overall HIV incidence reduction in 2020? Why did the authors only presented these data when explaining the incidence decrease in men?

LINES 242-245

The paragraph is confusing. The authors state that “the annual incidence of HIV among men has increased,” and then state that “the observed decline in HIV incidence.” Is this related to overall incidence (men and women)? If so, the sentence should be revised for clarity.

LINE 258

In the sentence, “Another group considered vulnerable are indigenous peoples, specifically from the Amazon regions of Peru such as Amazonas, Loreto and Ucayali,” the authors should use "is" instead of "are".
